# Comparison of the Effect of Vaginal *Zataria multiflora* Cream and Oral Metronidazole Pill on Results of Treatments for Vaginal Infections including Trichomoniasis and Bacterial Vaginosis in Women of Reproductive Age

**DOI:** 10.1155/2015/683640

**Published:** 2015-07-21

**Authors:** Khadijeh Abdali, Leila Jahed, Sedigheh Amooee, Mahnaz Zarshenas, Hamidreza Tabatabaee, Reza Bekhradi

**Affiliations:** ^1^Department of Midwifery, School of Nursing and Midwifery, Shiraz University of Medical Sciences, Shiraz 7193613119, Iran; ^2^Dezful Health Center, Dezful University of Medical Science, Dezful 7461715765, Iran; ^3^Faculty of Medicine, Shiraz University of Medical Sciences, Shiraz 713451978, Iran; ^4^Department of Statistics, Health Faculty, Shiraz University of Medical Sciences, Shiraz 713451978, Iran; ^5^Clinical Trials Department, Barij Essence Company, P.O. Box 1178, Kashan, Iran

## Abstract

Effect of* Zataria multiflora* on bacterial vaginosis and* Trichomonas vaginalis* is shown in vivo and in vitro. We compare the effectiveness of* Zataria multiflora* cream and oral metronidazole pill on results of treatment for vaginal infections including* Trichomonas* and bacterial vaginosis; these infections occur simultaneously. The study included 420 women with bacterial vaginosis,* Trichomonas vaginalis*, or both infections together, who were randomly divided into six groups. Criteria for diagnosis were wet smear and Gram stain. Vaginal* Zataria multiflora* cream and placebo pill were administered to the experiment groups; the control group received oral metronidazole pill and vaginal placebo cream. Comparison of the clinical symptoms showed no significant difference in all three vaginitis groups receiving metronidazole pill and vaginal* Zataria multiflora* cream. However, comparison of the wet smear test results was significant in patients with trichomoniasis and bacterial vaginosis associated with trichomoniasis in the two treatment groups (*p* = 0.001 and *p* = 0.01). Vaginal* Zataria multiflora* cream had the same effect of oral metronidazole tablets in improving clinical symptoms of all three vaginitis groups, as well as the treatment for bacterial vaginosis. It can be used as a drug for treatment of bacterial vaginosis and elimination of clinical symptoms of* Trichomonas* vaginitis.

## 1. Introduction

75% of women will experience vaginitis at least once in their lifetime. Most specialists believe that 90% of vaginitis cases are secondary to bacterial vaginosis, candidal vaginitis, and* Trichomonas vaginalis* [1].* Trichomonas vaginalis* is the most common gynecological diagnosis [2]. Several studies have known* Trichomonas* infection prevalence as variable.* Trichomonas vaginalis* is a flagellated protozoan that can be replaced in the human urogenital tract [3]. Exclusive diagnosis of* Trichomonas* can only be based on finding it in vaginal discharge [4].* Trichomonas* is usually transmitted sexually. In 90% of women infected with this protozoan, destruction occurs in the cervical region which can facilitate infection with a virus that can cause cancer [5].* Trichomonas vaginalis* can also be an important cofactor in HIV transfer [4]. In some studies,* Trichomonas vaginalis* has been known as a cause of infertility in both sexes [5].* Trichomonas vaginalis* is a proven cause of detachment of epithelial cells of the vagina and scaling of the mucosa in the clinical course [4].

*Trichomonas* vaginitis and bacterial vaginosis often exist simultaneously; in 60% of patients with this disease, bacterial vaginosis is also diagnosed [2]. BV is the most common type of vaginitis in women of childbearing age [6]. An American research reported the BV prevalence as 29.2% [7]. BV prevalence varies between 30 and 10% in different populations [6]. The golden BV diagnosing test is using a standard Nugent in a stained slide [8]. Women with BV are at high risk of pelvic inflammatory disease (PID), postabortion pelvic inflammatory disease, and infection of the vaginal cuff after hysterectomy and abnormal cytology of cervix (9, 2, and 10). Metronidazole is a drug of choice for treatment of* Trichomonas vaginalis* and bacterial vaginosis [2].

Although metronidazole is a selected treatment of* Trichomonas* and bacterial vaginosis, several reports from many parts of the world currently refer to the spread of resistance and its complications [9]. Side effects include nausea, vomiting, abdominal pain and uterine cramps, metallic taste in the mouth, vulvar itching, constipation, rash, swelling of the tongue, dizziness, fatigue, and darkening of the urine. In addition, metronidazole and its metabolites are mutagenic in bacteria and chronic administration induced tumor formation in rats [10]. In recent decades, numerous efforts have been made to select, extract, and find the mechanism of plant compounds against pathogens of sexually transmitted diseases (STDs) [11].* Zataria* is a familiar name for everyone. The term is used for a group of plants belonging to the Labiatae family including 200 genera and 3300 species [12, 13].* Zataria* genus has a particular species called* multiflora*. This plant has a limited distribution in the world [14].* Zataria* contains saponins, caffeic acid, resin, tannin, resonates, and finally 2.6% volatile oil referred to as essence. The most important components of this essence are thymol and carvacrol; these compounds have antimicrobial properties. Carvacrol is antiseptic and antifungal and thymol is antiseptic and antiworm [15, 16]. In a study,* Z. multiflora* essence could remarkably stimulate innate immunity function.* Z. multiflora* may be used to immunize alone or in combination with other immunostimulatory agents [17].

In a study, thyme properly influenced two organisms associated with leukorrhea (*Candida* and* Gardnerella*) [16]. Research has shown that vaginal douche by* Zataria multiflora* and clove extract can act as an anti-inflammatory [18]. Results of a study showed that vaginal* Zataria multiflora* cream was as effective as metronidazole vaginal gel in treating the symptoms of bacterial vaginosis [19]. In another study, the effect of 0.1% and 0.01%* Zataria multiflora* extract on* Trichomonas vaginalis* was at the beginning of the culture [20]. This study was conducted on trichomonatic secretions obtained from 100 patients with symptoms of vaginitis using methanol extract of* Artemisia*,* Zataria multiflora*, and* Myrtus*. Regarding the effect of the above plant extracts on* Trichomonas vaginalis*, it was suggested to examine the effect of the above plants individually or in a combination of three plants clinically on the parasites [20].

Despite the increasing use of herbal medicines in the treatment of vaginitis, side effects of drugs, increased prevalence of resistance and interference with other drugs as well as the demonstrated effect of* Zataria multiflora* on* Trichomonas vaginalis* and bacterial vaginosis in vitro, and the fact that 60% of* Trichomonas* infections are associated with bacterial vaginosis, little clinical research has been conducted on effects of vaginal* Zataria multiflora* cream on treatment of* Trichomonas* vaginal infections and bacterial vaginosis. There is no clinical study on the effect of vaginal* Zataria multiflora* cream on treatment of* Trichomonas* infection associated with vaginal BV. Therefore, this study examines the effect of vaginal* Zataria multiflora* cream on* Trichomonas* vaginal infections and bacterial vaginosis.

## 2. Materials and Methods

This study was a double-blind clinical trial to compare the effect of vaginal* Zataria multiflora* cream and oral metronidazole pill on results of treatment for* Trichomonas* vaginal infections, bacterial vaginosis, and both infections simultaneously, after approval by the Ethics Committee of Shiraz University of Medical Sciences. Using wet smear test and Gram stain, the target-based convenient sampling method was used to select 140 patients with* Trichomonas* vaginal infection, 140 patients with BV, and 140 patients with simultaneous* Trichomonas* vaginal infection and BV among qualified women of reproductive age with vaginitis symptoms referred to the Shiraz University of Medical Sciences. To determine the effect of treatment, women with the above infections were divided into two equal groups of experiment and control through block random allocation. This block randomization was conducted in the formulation sector of Barij Essence Pharmaceutical Company. Codes were kept by the formulation sector until the end of data collection. By this method, both randomization and double blinding were provided.

The experimental group was administered 0.1% vaginal* Zataria multiflora* cream, Leucorex, each tube containing 50 g and manufactured by Barij Essence Pharmaceutical Company for 7 nights and each night an applicator containing 5 g. The control group received 250 mg oral metronidazole tablet manufactured by Tehran Chemical Pharmaceutical Company, every 12 hours for seven days.

Inclusion criteria included (1) willingness to cooperate, (2) women of reproductive age, (3) wet samples positive for* Trichomonas*, (4) positive Gram stain for bacterial vaginosis, (5) wet samples positive for* Trichomonas* and Gram stain for BV, (6) symptoms of* Trichomonas* infection or signs of bacterial vaginosis in examination, (7) not taking medications to treat in the last three months, and (8) not taking antiparasitic drugs, antibiotics, immunosuppressive drugs, and vaginal medications.

Exclusion criteria included (1) abnormal uterine bleeding, (2) recurrent vulva vaginitis, (3) pregnancy, (4) menstruation, (5) breastfeeding, (6) frequent vaginal douche, (7) alcohol and coumarin anticoagulants, (8) multiple sexual partners, (9) specific diseases such as liver disease, kidney and central nervous system diseases, blood dyscrasia, diabetes, immune deficiency, and known venereal disease, (10) history of allergy to oral and topical Lamiaceae family, and (11) history of hypersensitivity to metronidazole tablets.

Using a sterile swab, secretions of the posterior vaginal cul and upper vaginal sidewall were sampled. A sample was sent to the laboratory immediately in 1 cc normal saline tube for wet smear test. A sample was placed and air-dried on a slide which was sent to the laboratory for Gram stain. By definitive diagnosis of bacterial vaginosis or* Trichomonas* or both, patients were allocated to the control or experiment groups. Experimental patients received 0.1%* Zataria multiflora* vaginal cream for one week; all patients took the medication only for one course, once a day. Patients were advised to lie in the supine position for at least 30 minutes after uptake. Moreover, subjects were given instructions for proper uptake of medication. Control patients received two 250 mg metronidazole tablets orally twice a day for one week. For blinded study, patients were administered placebo tablets for experiment group and vaginal placebo cream for control group. Spouses of the patients with* Trichomonas vaginalis* were also treated. The patients were also recommended to use condom if they had intercourse. The participants were asked to avoid other medications during the experiment. Seven days after the treatment ended, the procedures were redone. Untreated patients were recommended to take two 250 mg oral metronidazole tablets twice a day for one week after completion of treatment. The Wilcoxon, Mann-Whitney *U*, chi-square, and Fisher exact tests were used to analyze data.

## 3. Results

This study indicates the effect of* Zataria multiflora* vaginal cream to relieve clinical signs of bacterial vaginosis and trichomoniasis, as well as treatment of bacterial vaginosis. In this study, 2 patients due to lack of follow-up and 5 patients because of improper use of drugs were excluded from the study. Out of these patients, 4 belonged to the* Trichomonas* infection group and 3 belonged to the* Trichomonas* and bacterial vaginosis group.

Mann-Whitney test showed no significant differences in all three* Trichomonas*, bacterial vaginosis, and bacterial vaginosis-trichomoniasis, between control and experimental groups for age, number of pregnancies, and number of deliveries. Moreover, the chi-square test showed no significant difference between control and experiment groups for education.

Wilcoxon test showed that* Zataria multiflora* treatment significantly reduced clinical symptoms including foul-smelling discharge and itching in women with vaginal* Trichomonas* infection (*p* = 0.004, *p* = 0.007) and vaginal discharge, foul-smelling discharge, itching, and pain during intercourse in women with BV.

Mann-Whitney test showed that comparison of posttreatment clinical symptoms was not significant in both treatment groups for all clinical symptoms in three infection groups except for irritation in the* Trichomonas* infection (*p* = 0.006) and irritation and dysuria in the BV-*Trichomonas* infection (*p* = 0.035, *p* = 0.034).

To compare posttreatment results in two groups, chi-square test and Fisher's tests were used. Comparison of results was not significant for Gram stain of bacterial vaginosis in the two treatment groups (*p* = 0.221, Table 1), while it was significant for wet smear test of* Trichomonas* in the two treatment groups (*p* = 0.001, Table 2). For bacterial vaginosis-trichomoniasis group, comparison of wet smear result was significant (*p* = 0.01), while it was not significant for Gram stain test (*p* = 0.72, Table 3).

Chi-square and Fisher's exact tests showed that the percentage emergence of side effects is higher in the metronidazole pill group (metallic taste in the mouth, nausea, and dizziness) (*p* < 0.001, *p* < 0.001, and *p* < 0.008) and the* Zataria multiflora* group (irritation) (*p* < 0.002).

## 4. Discussion and Conclusions

*Zataria multiflora* essence contains 69% phenol and often carvacrol; its main nonphenolic element is P-cymene. The main compounds of this essence include thymol and carvacrol followed by linalool and P-cymene [21]. Carvacrol is the main component of* Zataria multiflora* essence whose antibacterial and antifungal activity has been demonstrated on different microorganisms. Hydrophobic compounds such as carvacrol influence biological membranes of microorganisms [22].

Analgesic effects of* Zataria multiflora* have been shown on chronic pains of rats [23].* Zataria multiflora* has an antispasmodic effect on smooth muscles, which is used in treatment of irritable bowel syndrome (IBS). This plant is also antitussive. Thymol and carvacrol as the main components of essence have good antimicrobial effects.* Zataria multiflora* is a good digestive stimulator and it is helpful for people with digestive problems and during convalescence [12, 24].

The results of this study suggest that vaginal* Zataria multiflora* cream alleviates the symptoms of foul-smelling discharge and itching (*p* = 0.0004, *p* = 0.007) in women with* Trichomonas* vaginitis and clinical symptoms of vaginal discharge, foul-smelling discharge, itching, and pain during intercourse in women with bacterial vaginosis.

To evaluate the effect of* Zataria multiflora* vaginal cream and metronidazole gel on bacterial vaginosis, Simbar et al. [19] examined the clinical symptoms of vaginal discharge, foul-smelling discharge, painful intercourse, abdominal pain, itching, and dysuria. This study found significant differences between pre- and posttreatments with* Zataria multiflora* cream to relieve symptoms except itching (McNemar test, *p* = 0.01). Although complaints about itching reduced from 16.3% to 4.7% before and after the treatment by* Zataria multiflora* cream, the reduction was not significant; however, the difference was significant before and after the treatment by metronidazole gel (*p* < 0.01). In the current study, posttreatment clinical symptoms of both* Zataria multiflora* and metronidazole showed no significant difference in improved clinical symptoms of BV; therefore, current findings are consistent with Simbar et al. [19]. It is shown that the antimicrobial activity of essences is related to their phenolic compounds. Antibacterial effect of* Zataria multiflora* is also related to phenolic compounds, especially carvacrol and thymol [24].

For a clinical comparison of vaginal douche by* Zataria multiflora* clove extracts in inflammatory vaginitis, Mucci [18] found a significant reduction in symptoms of vaginal discharge, itching, dysuria, and pain during intercourse after treatment (*p* < 0.01). Improvement of these clinical signs was attributed to anti-inflammatory, antioxidant, and antibacterial activity of* Zataria multiflora* and clove extracts as well as the effect of these plants in reducing the adhesion and penetration of bacteria into the cells of the vagina. This is consistent with findings of this study.* Zataria multiflora* extract can reduce adhesion of bacteria to the vaginal wall cells at lower concentrations below the minimum inhibitory concentration [24, 25].

Comparing the effects of vaginal* Zataria multiflora* cream and clotrimazole on treatment of acute vaginal candidiasis, Khosravi et al. [26] found that* Zataria multiflora* was able to reduce the symptoms of vaginal discharge, itching, dysuria, and dyspareunia considerably (*p* < 0.05), and the effect was similar to that of clotrimazole. These findings are also consistent with this study. Effect of* Zataria multiflora* on clinical improvement can be attributed to carvacrol. Carvacrol affects the biomembrane of microorganisms and its antibacterial and antifungal effects have been demonstrated.

To compare the effectiveness of vaginal thymol and clove gel and econazole to treat bacterial vaginosis, De leo and Benvernuti [24] examined the clinical symptoms of itching, irritation, and vaginal discharge which significantly decreased after treatment (*p* = 0.0002).

The present study supports findings by Simbar et al. [19], Khosravi et al. [26], and Niak [23] on clinical improvement of foul-smelling discharge, itching, and pain during intercourse, after treatment by* Zataria multiflora*. Although complaints about itching and dysuria reduced after treatment by* Zataria multiflora* cream in three vaginitis groups, the reduction was not significant. Effect of metronidazole was insignificant on these two symptoms of the BV group (*p* = 0.217, *p* = 0.172). Perhaps the reason for nonimproved dysuria is urinary tract infection which existed with the studied vaginitis. However, urinary infection was out of the scope of this study and requires more studies. The person feels irritation in the reproductive tract, being close to the urethra, if any exists in the urethra. A common cause of vaginal irritation is due to scratching caused by infections such as yeast infections, chlamydia, and gonorrhea in the genital tract. Vaginal irritation can also be caused by diseases and other disorders like hormonal change, allergic reactions to substances such as scented soap and tampons, vulvar dystrophy, lichen sclerosis, intercourse, and physical and mechanical damage. This study did not examine all causes of vaginal irritation; this requires more studies by controlling the above factors.

Comparing* Zataria multiflora* vaginal cream to oral metronidazole pill, we note that vaginal cream acts as metronidazole to relieve clinical symptoms, except for irritation in the* Trichomonas* infection and irritation and dysuria in the bacterial vaginosis-trichomoniasis.

To compare the effect of* Zataria multiflora* vaginal cream and oral metronidazole pill on treatment of vaginal* Trichomonas* infection, Glomakani et al. [27] examined clinical symptoms of leucorrhea, itching, irritation, dysuria, strawberry cervix, pain during intercourse, and patchy erythema. In this study, the successful clinical outcomes were obtained for 88.9% of the subjects treated by* Zataria multiflora* and 63% of individuals treated by metronidazole (*p* = 0.026) and improvement of clinical symptoms was higher in the* Zataria multiflora* group than the metronidazole group. This was attributed to anti-inflammatory effect of* Zataria multiflora*. In this study, anti-inflammatory effect of* Zataria multiflora* to relieve symptoms causes an effect similar to oral metronidazole tablets.

Results of this study also showed that posttreatment Gram stain test obtained similar results for both treatment groups (oral metronidazole and vaginal* Zataria multiflora*) in bacterial vaginosis and BV-trichomoniasis (*p* = 0.221, *p* = 0.72). These two drugs had similar effects on treatment of BV. Results of this study are similar to Simbar. However, the wet smear test provided different results for trichomoniasis infection and BV-trichomoniasis (*p* = 0.01, *p* = 0.001). Golmakani achieved therapeutic success by wet smear in 51.9% of the* Zataria multiflora* subjects and 889% of the metronidazole group (*p* < 0.003). He concluded that therapeutic success of* Zataria multiflora* vaginal cream is lower than the metronidazole pill. The findings of this study show that negative posttreatment wet smear test was 71.43% in women with trichomoniasis and 74.29% in women with bacterial vaginosis-trichomoniasis treated by* Zataria multiflora* and 92.86% and 91.43%, respectively, treated by oral metronidazole. There are significant differences between metronidazole pill and* Zataria multiflora* vaginal cream (*p* = 0.01, *p* = 0.001), which indicates that success rate of vaginal cream is lower than metronidazole. This is consistent with Golmakani; however, negative posttreatment wet smear test was higher in the present study. This can be attributed to proper use of medications, use of condoms during treatment, and treatment of spouses. It must be considered that* Zataria multiflora* vaginal cream is topical. A longer time may be required after the end of treatment to take wet smears from patients in order to determine the effect of* Zataria multiflora* on treatment of vaginitis. This study ignored the effect of* Zataria multiflora* on recurrence of* Trichomonas* vaginitis; further studies are recommended for this purpose.

De leo and Benvernuti [24] showed that thymol and clove vaginal gel was as effective as econazole when there is only bacterial vaginosis; in combination with other infections, econazole is more effective (*p* < 0.01). In the present study,* Zataria multiflora* was as effective as metronidazole in improving the clinical symptoms of* Trichomonas vaginalis*-BV; it also acted as metronidazole in treating BV (*p* = 0.72). Only metronidazole was more effective in treating* Trichomonas* (*p* = 0.01). De leo and Benvernuti [24] only used thymol vaginal gel, while the effect of* Zataria multiflora* in improving clinical symptoms of* Trichomonas vaginalis*-BV can be attributed to the carvacrol existing in* Zataria multiflora* because the biological activity of the* Z. multiflora* is mainly related to the phenolic compounds existing in* Z. multiflora*, such as thymol and carvacrol. However, Hadian et al. [28] showed that the knowledge on diversity of the* Z. multiflora* chemotype, illustrated in this study, will allow an improvement of the homogeneity of the plant material for production of different types of essential oils, depending on specific uses in pharmaceutical and food industries. On the other hand, some studies showed that* Z. multiflora* extract is antioxidant and antibacterial [25] and can reduce the adhesion of bacteria to the vaginal wall cells at concentrations lower than the inhibitory concentration [26]. Existing evidence requires further experimental and clinical investigations on the effect of* Z. multiflora* and its materials on* T. vaginalis*.

The reported side effects included irritation when using the vaginal cream (9 subjects using* Zataria multiflora* vaginal cream), metallic taste in mouth, nausea, dizziness, and vomiting (24, 15, 8, and 3 subjects, resp., using metronidazole pill).

Khosravi et al. [26] showed that 100% of people using* Zataria multiflora* and 51% of the subjects using metronidazole experienced no side effects. There was a significant difference in side effects between these two groups (*p* < 0.0001), which is inconsistent with the present study. In the present study,* Zataria multiflora* was associated with vaginal irritation; thymol existing in* Zataria multiflora*, which can sometimes cause mucosal irritation, may cause vaginal irritation. However, Golmakani studied a lower sample size (27 subjects). As noted earlier, factors such as vaginal yeast infections, gonorrhea, reduced levels of body estradiol, intercourse, and mechanical injuries can cause vaginal irritation, which are not studied here. In Mucci [18], metronidazole caused side effects of dizziness, bad metallic taste, nausea, and vaginal dryness and* Zataria multiflora* caused vaginal dryness and nausea similar to metronidazole and vaginal irritation more than metronidazole, while the present study showed that metronidazole group experienced more bad metallic taste, dizziness, and nausea and the* Zataria multiflora* group experienced more vaginal irritation. In conclusion, this study showed that clinical improvement was similar in all three groups,* Trichomonas* vaginitis, bacterial vaginosis, and bacterial vaginosis-trichomoniasis, using oral metronidazole tablets and* Zataria multiflora* vaginal cream. In addition, the* Zataria multiflora* vaginal cream was as effective as metronidazole on treatment of bacterial vaginosis in both groups of bacterial vaginosis and bacterial vaginosis-trichomoniasis. Man is a part of nature; nature cures every disease by plants available to him. This is why when man turns to nature to treat his disease he is treated faster, better, and more safely. Effective ingredients of herbal medicines with other materials are biologically balanced; therefore, they do not accumulate in the body and do not cause side effects. Hence, they are considerably advantageous over chemical drugs.* Zataria multiflora* is an herbal medicine from herbal flora of Iran. Our findings indicate that* Zataria multiflora* vaginal cream is as effective as metronidazole in the treatment of bacterial vaginosis and clinical symptoms of* Trichomonas* vaginitis and bacterial vaginosis-trichomoniasis. Therefore, the medical staff can prescribe* Zataria multiflora* vaginal cream to treat bacterial vaginosis and relieve the clinical symptoms of trichomoniasis. However, this study ignored the effect of* Zataria multiflora* on recurrence of* Trichomonas* vaginitis; further studies are recommended for this purpose.

## Figures and Tables

**Table 1:** Comparison of posttreatment results for women with BV treated by *Zataria multiflora* and metronidazole.

Posttreatment result	Drug				
	Oral metronidazole pill		Vaginal *Zataria multiflora* cream		Sig.
	Number	%	Number	%	
Gram stain					
Negative	68	97.1	65	92.9	0.221
Positive	2	2.9	5	7.1	
Total	70	100	70	100	

**Table 2:** Comparison of posttreatment results for women with trichomoniasis treated by *Zataria multiflora* and metronidazole.

Posttreatment result	Drug				
	Oral metronidazole pill		Vaginal *Zataria multiflora* cream		Sig.
	Number	%	Number	%	
Wet smear					
Negative	65	92.86	50	71.43	0.001
Positive	3	4.28	18	25.71	
Eliminated	2	2.86	2	2.86	
Total	70	100	70	100	

**Table 3:** Comparison of posttreatment results for women with BV-trichomoniasis treated by *Zataria multiflora* and metronidazole.

Posttreatment result	Drug				
	Oral metronidazole pill		Vaginal *Zataria multiflora* cream		Sig.
	Number	%	Number	%	
Wet smear					
Negative	64	91.4	52	74.29	0.01
Positive	5	7.1	16	22.85	
Eliminated	1	1.4	2	2.86	
Total	70	100	70	100	

Gram stain					
Negative	66	94.3	64	91.43	00.72
Positive	3	4.3	4	5.71	
Eliminated	1	1.4	2	2.86	
Total	70	100	70	100	
